# A three years retrospective study on the nature and cause of ocular dermoids in cross-bred calves

**Published:** 2012-04-17

**Authors:** M.M. Alam, M.M. Rahman

**Affiliations:** 1 *Department of Surgery and Obstetrics, Bangladesh Agricultural University, Mymensingh, Bangladesh*; 2 *Upazila Veterinary Surgeon, Durgapur, Rajshahi, Bangladesh*

**Keywords:** Cross-bred calves, Dermoid cysts, Incidence, Ocular dermoids, Surgical intervention

## Abstract

Nature and cause of ocular dermoids were investigated by field studies, pedigree analysis, clinical examination and light microscopy. It was determined that ocular dermoids in cross-bred calves are genetically-transmitted defects. Calves typically were affected unilaterally or bilaterally with multiple, connected ocular growths that clinically and histologically mimicked normal haired skin. Sites most commonly involved included ventro-lateral limbus, third eyelid, medial canthus, eyelid and conjunctiva. Centro-corneal and anterior segmental dermoids were also observed. It is postulated from this study that bilateral ocular dermoids are genetically-transmitted defects in Hereford cattle. Characteristics of both autosomal recessive and polygenic inheritance were observed. However, mode of inheritance and role of environment in the pathogenesis of these peculiar and important developmental defects remain undefined. Extensive breeding trials utilizing superovulation, embryo transfer, and pre-terminal caesarean section as well as further field studies might be mandatory to confirm sex incidence, significance of associated somatic defects, role of environment in phenotypic expression, and mode of transmission of ocular dermoids in cross-bred calves.

## Introduction

Dermoid cyst is an uncommon developmental anomaly that has been reported in dogs, cats, horses, and cattle (Lawson, 1975). The cyst is usually congenital or hereditary in nature (Adams *et al.*, 1983). It may be solitary or multiple, firm to fluctuant, well circumscribed, smooth, and round and usually the overlaying skin is normal (Shields *et al.*, 1986). Dermoid cysts are formed due to defective epidermal closure along embryonic fissures, which isolates an island of ectoderm in the dermis or subcutis. The cyst usually contains hair, keratin, and sebum, and these materials may produce progressive enlargement of the structure so that it becomes clinically apparent (Edwards, 2002).

Histologically, dermoid cysts/sinuses are lined with stratified epithelium resembling normal skin with adnexa and filled with keratinous material (Munoz *et al.*, 2007). The cyst occurs most commonly in the horse, especially on the dorsal midline, between the withers and the rump (Gordon, 1978; Mullowney, 1985). Other common locations of these cysts in the horse are the base of the ear, as dentigerous cysts, or in the false nostril, as atheromas (Hillyer *et al.*, 2003). There is one case reported of retrobulbar dermoid cyst in an Andalusian horse (Munoz *et al.*, 2007).

In the young dogs, the cutaneous dermoid cysts are most often seen in the Rhodesian Ridgeback, Siberian Husky, Shih Tzu, Boxer, and Kerry Blue terrier breeds. In most cases, dermoid cysts in dogs are associated with multiple vertebral and spinal malformations and hind limb neurologic deficits (Bhatt *et al.*, 1964).

Cutaneous or subcutaneous cysts of all types are considered rare in cats, and the two previously reported cases were seen in domestic shorthair cat (Baird *et al.*, 1993). Ocular dermoids are rarely reported in calves (Shields *et al.*, 1986).

The purpose of this study was to enrich the literature with fifty four cases of ocular dermoids which caused moderate to marked disfigurement of external ocular structures.

## Materials and Methods

### History

Fifty four calves with congenital ocular dermoids were presented in the veterinary clinic of Bangladesh Agricultural University (BAUVC) from 2006-2008 (Table 1).

**Table 1:** Occurrence in year, breed, sex, and involvement in eyes of 54 calves with ocular dermoids

Year	Calves with dermoids	Sex		Involvement		

		M	F	Uni.	Bi.	Unknown
2006	17	10	7	3	14	0
2007	16	9	7	4	12	0
2008	21	11	10	7	11	3
Total	54	30	24	14	37	3

Uni. = Unilateral, Bi. = Bilateral.

History of adverse environmental factors associated with these cases was asked from the owner. The following factors were investigated: breed, sex, degree of ocular involvement, age of parents, geographic region, season, type of pasture, soil type, exposure to or suspected exposure to teratogenic plants, feeding and management practices, breeding records, maternal medical and vaccination records, disease status of the herd, periods of stress, drugs administered, congenital defects observed previously, and history of similar congenital defects in neighboring herds. Breeding records of defective calves were collected.

### Clinical examination

The corneal dermoids extended slightly beyond the inferonasal limbus and then merged with a second mass of lightly haired tissue within the inferonasal bulbar conjunctiva of both eyes. The nictitans of both eyes was poorly developed, the left nictitans being particularly rudimentary.

### Surgical procedure

After clinical examination, corneal dermoids were resected after mild sedation with xylazine hydrochloride (Rompun®, Bayer, Germany) at 0.1 mg/kg IM. Local infiltration with 2% lignocaine hydrochloride (Jasocaine, Jayson Pharmaceuticals, Dhaka, Bangladesh) was also performed. Local anaesthetic was infiltrated directly into the stalk which facilitated separation of dermis from cornea. Auriculopalpebral block was not necessary.

The dermoids were excised by superficial lamellar keratectomy that was extended into the inferonasal conjunctiva. All excised tissue was submitted for histopathology. Cysts were carefully dissected from all adherent areas, stalks were ligated with chromic catgut (1.0). Postoperatively, systemic penicillin and streptomycin were administered IM in addition to a local antibiotic (for eye instillation).

Ocular tissues were fixed in 10% neutral buffered formalin, embedded in paraffin, sectioned at 5 µm by a sliding microtome, and stained with hematoxylin and eosin (HE).

## Results

References to the fifty four calves with congenital ocular dermoids were reported in the present study (Table 1). Breeding history revealed calves were graded as cross-bred (Holstein Friesian x local). No other cattle breeds were found in this study. Thirty calves were males and twenty four females.

Parents were phenotypically normal except one bull and one cow. The bull had a thickened nodular third eyelid and the cow had a small speck involving the cornea of one eye.

Thirty seven calves had dermoid growths affecting both eyes, fourteen were unilateral, and involvement of three was not reported. Calves were between three to fourteen months old and operation was mostly carried out after proper inspection. There was marked variation in the extent of dermoids, which ranged from a small growth to marked ocular malformation with blindness. The blindness was due to irritation leading to corneal opacity.

There were no environmental factors associated with the ocular dermoids. One reference indicated vitamin A deficiency 10 years earlier and except for infectious keratoconjunctivitis in one case, no significant viral or bacterial diseases were reported.

The use of drugs or other medications in cows having calves with dermoids was not reported and administration of vaccines was according to the routinely vaccination schedules.

Solanum spp (Nightshade) was found to be a problem in one case; a survey of the grazing areas of other herds, however, did not reveal any poisonous plants (data not shown).

Breeding history indicated that 22 dermoids (15 male, 7 female) had separate dams and were sired by seven different bulls; 15 calves were sired by one bull. The sire of this bull also sired a calf with a dermoid and a grandsire of a bull reported to have sired a calf with a dermoid. Three defective calves were sired by a common bull, and the remaining three bulls sired one defective calf each.

There was considerable variation among individual eyes and between calves in expression of the defect depending on location, size, amount and type of hair, and number of skin-like masses present. Most calves were affected bilaterally with multiple, connected ocular dermoids resulting in mild to marked disfigurement of cornea, canthi, and eyelids.

There was encroachment on the visual axis with various degrees of visual impairment, and significant subacute keratoconjunctivitis with epiphora, blepharospasm, and corneal ulceration. Inflammation was related directly to irritation by the dermoid and to exposure keratitis secondary to eyelid dysfunction. Sites most commonly affected included limbus, third eyelid, canthus, eyelid, and conjunctiva (Table 2).

**Table 2:** Dermoid cyst involvement in both eyes

Eye	A.S.	C.C.	L.	C.	T.E.	Ca.	E.
Right	1	8	1	3	2	18	4
Left	2	14	2	3	2	15	3

A.S. = Anterior segment, C.C. = Central cornea, L. = Limbus, C. = Conjunctiva, T.E. = Third eyelid, Ca. = Canthus, E. = Eyelid.

Dermoids strictly of lacrimal caruncle and dermolipomas of the conjunctiva were not observed. Variation within and among the numerous dermoids observed histologically and included epidermal thickness, degree of epidermal keratinization and pigmentation, dermal papillae, degree of subacute subepidermal inflammation, number of epidermal adnexa, dermal thickness, amount of adipose tissue in subcutis, and depth of corneal replacement.

Subacute subepidermal, conjunctival, and corneal inflammation were observed in all dermoids and adherent intradermal lacrimal tissue frequently was present in deeper dermoid layers. Anterior segmental dermoids contained fascicles of skeletal muscle and bands of hyaline cartilage. No postoperative complications were noticed.

**Fig. 1:**
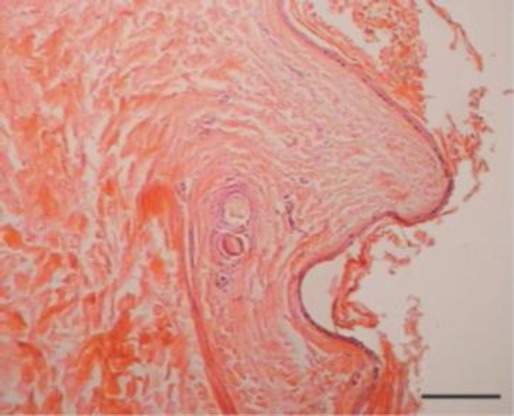
The cyst is lined by a stratified squamous epithelium, and its lumen is filled with squamous debris and desquamated keratin (H&E). Bar = 200 μm.

Histopathology was similar for the tissue excised from both eyes. The corneal lesion exhibited a moderately well delineated but non-encapsulated raised mass composed of moderately hyperplastic, keratinized stratified squamous epithelium overlying a thick collagenous stroma, which merged with conjunctival tissue containing submucosal glandular tissue (Fig. 1). The corneal mass contained numerous, large, well developed hair follicles and adnexal structures superficially.

A band of abortive hair follicles and adnexa was identified as haphazardly arranged clusters of epithelium, in the absence of hair bulbs, intermingled with tortuous lumina of apocrine glands beneath the productive follicles. This was accompanied by myxomatous stroma and a minimal neutrophilic and eosinophilic inflammatory infiltrate.

**Fig. 2:**
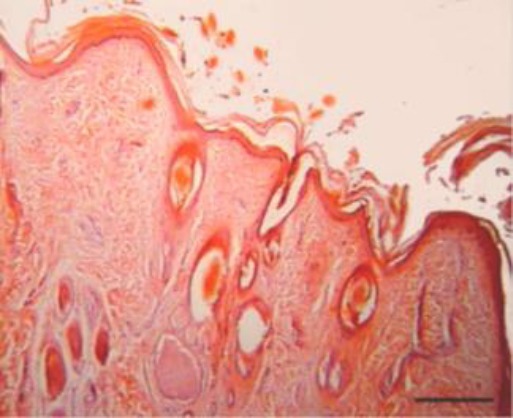
Superficial aspect of dermoid cyst with the wall consisted of three to ten layers of cells, and widely scattered adnexal structures are associated with the cyst wall (H&E). Bar = 330 μm.

Histopathology of the excised nasal tissue was similar and characterized by moderately well delineated but nonencapsulatedraised masses. These were composed of moderately hyperplastic, keratinized stratified squamous epithelium overlying large regularly arranged hair follicles and adnexa (Fig. 2).

Clusters of compound tubulo-alveolar glands showing moderate mitotic activity were located deep to the base of the hair follicles. Morphologic diagnosis was bilateral corneoconjunctival dermoids with ectopic lacrimal glands, and bilateral nasal choristomas with ectopic nasal vestibular glands.

## Discussion

The precise developmental mechanisms involved in the pathogenesis of ocular dermoids are not known. Metaplasia of mesenchyme (of primarily neural crest origin), resulting in abnormal differentiation of the surface ectoderm, is considered the most likely mechanism. The resulting dermoid consists of ectodermal elements (keratinized epithelium, hairs, sebaceous and apocrine glands), and mesenchymal elements (fibrous tissue, fat and cartilage) combined in different proportions.

Ocular dermoids in cattle are not common, with an estimated prevalence of 0.002%–0.4% in USA (Greene *et al.*, 1973). Ocular dermoids have been reported in cattle of many breeds worldwide, with a similar low prevalence in all breeds other than the Hereford (Barkyoumb and Leipold, 1984). The dermoid locations in this study were reported in decreasing order as limbus, third eyelid, canthus, eyelid and conjunctiva.

There are otherwise few reports of bilateral ocular dermoids in calves, each describing single or low numbers of animals, and only one reporting a nasal tumor-like growth. Of these bilateral cases, inferonasal corneoconjunctival dermoids were most commonly reported, followed by nasal canthal dermoids.

Ocular dermoids may be associated with other congenital ocular or multi-organ abnormalities (Mansour *et al.*, 1989). The medial and lateral nasal processes, optic vesicle and first and second branchial arches are in close relationship on the lateral side of the embryonic head and might all be adversely influenced by the same stimulus.

The combination of congenital ocular and nasal abnormalities in calf is consistent with the intimate early developmental origin of the optic and nasal regions and a common abnormality in neural crest migration; whether this abnormality has a genetic basis or not is less clear.

Characteristics of both autosomal recessive and polygenic inheritance were found by (Barkyoumb and Leipold, 1984,) in their analysis of Hereford cattle with ocular dermoids.

Breed predispositions for ocular dermoids were reported in Birman cats, Dachshund, Dalmatian, Dobermann, Golden Retriever, German shepherd and Saint Bernard dogs, and Quarter horses.

Other than the autosomally-dominant ring dermoid syndrome, which presents as bilateral corneal dermoids involving the limbus for 360 degrees with no other anomalies, neither corneal dermoid nor Goldenhar’s syndrome is considered to be genetic in humans.

Dermoids have been attributed to metaplasia of corneal epithelium secondary to excessive exposure of the cornea to the intrauterine environment caused by abnormal development and closure of the eyelid (Shields *et al.*, 1986).

Other causes are adhesion and implantation of portions of amnion, (Lawson, 1975), attachment of eyelids to the corneal surface (Bhatt *et al.*, 1964), invagination of isolated islands of surface ectoderm capable of developing into skin and adnexa and abnormal differentiation of mesoderm lying between the optic cup and surface ectoderm (Barkyoumb and Leipold, 1984)

Corneal formation begins when the lens vesicle closes with the remaining surface ectoderm overlying the mouth of the optic cup giving rise to anterior corneal epithelium and its basal lamina.

It is at this time that mesenchymal cells surrounding the optic cup migrate between the anterior surface of the lens vesicle and overlying anterior corneal epithelium. Infiltration of corneogenic tissues occurs in waves with the first wave resulting in formation of posterior corneal endothelium. Successive waves of invading mesenchyma form the substantia propria of the cornea, first posteriorly, then anteriorly (Ismail, 1993).

High incidence in cross-bred calves of bilateral expression of the defect, common ancestry suggesting familial tendencies, occurrence in males more than females, independence from season and inability to demonstrate any known teratogenic environmental influence suggested that ocular dermoids in cattle observed in this study may be transmitted genetically (Castro *et al.*, 2006).

It is postulated from this study that bilateral ocular dermoids are genetically transmitted defects in Hereford cattle.

Characteristics of both autosomal recessive and polygenic inheritance were observed (Oakman *et al.*, 1993). However, mode of inheritance and role of environment in the pathogenesis of these peculiar and important developmental defects remain undefined. Extensive breeding trials utilizing superovulation, embryo transfer, and preterminal caesarean section as well as further field studies will be necessary to confirm sex incidence, significance of associated somatic defects, role of environment in phenotypic expression, and mode of transmission of ocular dermoids in cross-bred calves.

The present study, which revealed the nature and cause of dermoids in cross-bred calves, will be useful for the veterinary surgeon for promptly approaching the indicated patients.
